# Supplementary material for: How do clinician and parent-reported data differ? An analysis of similarity and difference in the datasets from a cross-syndrome genetics cohort study (GenROC)
Source: J Med Genet. 2026 Jan 19;63(4):e111193. doi: 10.1136/jmg-2025-111193 (PMC13018764; doi:10.1136/jmg-2025-111193)
Supplement: online supplemental table 1 [file jmg-63-4-s001.docx]

| **Supplemental Table1.** | **Gene group categories with counts** | |
| --- | --- | --- |
| **Genes** | **Category** | **Count** |
| **Chromatin related n=124** | | |
| ANKRD11 | Chromatin related | 28 |
| ADNP | Chromatin related | 11 |
| ASH1L | Chromatin related | 4 |
| ASXL3 | Chromatin related | 5 |
| BPTF | Chromatin related | 2 |
| BRWD3 | Chromatin related | 1 |
| CHD2 | Chromatin related | 6 |
| CHD3 | Chromatin related | 6 |
| CHD4 | Chromatin related | 4 |
| CHD7 | Chromatin related | 5 |
| CHD8 | Chromatin related | 5 |
| KANSL1 | Chromatin related | 2 |
| KAT6A | Chromatin related | 3 |
| KAT6B | Chromatin related | 2 |
| KDM5B | Chromatin related | 2 |
| KDM5C | Chromatin related | 3 |
| KMT2A | Chromatin related | 12 |
| KMT2C | Chromatin related | 2 |
| KMT5B | Chromatin related | 1 |
| MECP2 | Chromatin related | 8 |
| NSD2 | Chromatin related | 1 |
| SETD5 | Chromatin related | 8 |
| SRCAP | Chromatin related | 3 |
| **Cytoskeletal n = 44** | | |
| ACTB | Cytoskeletal | 3 |
| CNTNAP1 | Cytoskeletal | 2 |
| DYNC1H1 | Cytoskeletal | 4 |
| KIF1A | Cytoskeletal | 10 |
| NRXN1 | Cytoskeletal | 3 |
| SMC1A | Cytoskeletal | 2 |
| SMC3 | Cytoskeletal | 3 |
| SPTAN1 | Cytoskeletal | 1 |
| TUBA1A | Cytoskeletal | 14 |
| CLTC | Cytoskeletal | 2 |
| **Gene specific transcriptional regulator N =99** | | |
| ATRX | Gene specific transcriptional regulator | 6 |
| BCL11A | Gene specific transcriptional regulator | 2 |
| BRPF1 | Gene specific transcriptional regulator | 3 |
| CAMTA1 | Gene specific transcriptional regulator | 2 |
| CTNNB1 | Gene specific transcriptional regulator | 10 |
| DEAF1 | Gene specific transcriptional regulator | 3 |
| EBF3 | Gene specific transcriptional regulator | 8 |
| ERF | Gene specific transcriptional regulator | 1 |
| FOXG1 | Gene specific transcriptional regulator | 2 |
| FOXP1 | Gene specific transcriptional regulator | 6 |
| GATAD2B | Gene specific transcriptional regulator | 6 |
| GLI2 | Gene specific transcriptional regulator | 2 |
| MED13L | Gene specific transcriptional regulator | 7 |
| MEF2C | Gene specific transcriptional regulator | 3 |
| NFIX | Gene specific transcriptional regulator | 5 |
| POGZ | Gene specific transcriptional regulator | 4 |
| PURA | Gene specific transcriptional regulator | 3 |
| RAI1 | Gene specific transcriptional regulator | 4 |
| SATB2 | Gene specific transcriptional regulator | 7 |
| TCF20 | Gene specific transcriptional regulator | 2 |
| TCF4 | Gene specific transcriptional regulator | 9 |
| ZBTB20 | Gene specific transcriptional regulator | 1 |
| ZMYND11 | Gene specific transcriptional regulator | 3 |
| **Glutamate channel n=20** | | |
| GRIK2 | Glutamate channel | 1 |
| GRIN1 | Glutamate channel | 2 |
| GRIN2A | Glutamate channel | 5 |
| GRIN2B | Glutamate channel | 12 |
| **Protein binding activity n =68** | | |
| EEF1A2 | Protein binding activity | 1 |
| FBX11 | Protein binding activity | 1 |
| IQSEC1 | Protein binding activity | 1 |
| OPHN1 | Protein binding activity | 3 |
| SYNGAP1 | Protein binding activity | 8 |
| TRIO | Protein binding activity | 8 |
| ARSA | Protein modifying enzyme | 1 |
| CDK13 | Protein modifying enzyme | 8 |
| DYRK1A | Protein modifying enzyme | 10 |
| HECW2 | Protein modifying enzyme | 1 |
| HUWE1 | Protein modifying enzyme | 5 |
| PPP2R5D | Protein modifying enzyme | 7 |
| TLK2 | Protein modifying enzyme | 7 |
| TRIP12 | Protein modifying enzyme | 3 |
| USP9X | Protein modifying enzyme | 4 |
| **RNA metabolism n=39** | | |
| CNOT3 | RNA metabolism | 3 |
| DDX3X | RNA metabolism | 11 |
| EFTUD2 | RNA metabolism | 3 |
| HNRNPU | RNA metabolism | 2 |
| MED12 | RNA metabolism | 1 |
| PUF60 | RNA metabolism | 9 |
| RNU42 | RNA metabolism | 7 |
| SON | RNA metabolism | 2 |
| TAF1 | RNA metabolism | 1 |
| **Scaffold adaptor n= 42** | | |
| CASK | Scaffold adaptor | 11 |
| MAGEL2 | Scaffold adaptor | 1 |
| SHANK2 | Scaffold adaptor | 3 |
| SHANK3 | Scaffold adaptor | 3 |
| TRAPPC9 | Scaffold adaptor | 1 |
| WAC | Scaffold adaptor | 7 |
| WDR45 | Scaffold adaptor | 2 |
| WDR62 | Scaffold adaptor | 1 |
| STXBP1 | Scaffold adaptor | 12 |
| WDR73 | Scaffold adaptor | 1 |
| **Transcriptional regulator n= 33** | | |
| AHDC1 | Transcriptional regulator | 3 |
| AUTS2 | Transcriptional regulator | 2 |
| CHAMP1 | Transcriptional regulator | 1 |
| CSNK2A1 | Transcriptional regulator | 4 |
| FOX2B | Transcriptional regulator | 1 |
| MED13L | Transcriptional regulator | 7 |
| MYT1L | Transcriptional regulator | 3 |
| PACS1 | Transcriptional regulator | 5 |
| SOX5 | Transcriptional regulator | 6 |
| TBL1XR1 | Transcriptional regulator | 1 |
| **Transporter n = 72** | | |
| ATP1A3 | Transporter | 8 |
| CACNA1A | Transporter | 11 |
| ITPR1 | Transporter | 2 |
| KCNQ2 | Transporter | 6 |
| NALCN | Transporter | 1 |
| SCN1A | Transporter | 7 |
| SCN1B | Transporter | 7 |
| SCN2A | Transporter | 11 |
| SCN8A | Transporter | 8 |
| SLC6A1 | Transporter | 6 |
| SLC6A8 | Transporter | 2 |
| VPS13B | Transporter | 3 |
| **Unclassified n=6** | |  |
| CTCF |  | 3 |
| NAA10 |  | 1 |
| NAA15 |  | 1 |
| NEXMIF |  | 1 |
| **Total** |  | **547** |
